# Clinical Evaluation of Dental Implants with a Double Acid-Etched Surface Treatment: A Cohort Observational Study with Up to 10-Year Follow-Up

**DOI:** 10.3390/ma14216483

**Published:** 2021-10-28

**Authors:** Juan Santos Marino, Jorge Cortés-Bretón Brinkmann, Ignacio García-Gil, Natalia Martínez-Rodríguez, Javier Flores Fraile, Cristina Barona Dorado, José María Martínez-González

**Affiliations:** 1 Department of Surgery, Faculty of Medicine, University of Salamanca, 37007 Salamanca, Spain; juan_santos_marino@hotmail.com (J.S.M.); j.flores@usal.es (J.F.F.); 2 Department of dental Clinical Specialties, Faculty of dentistry, Complutense University of Madrid, 28040 Madrid, Spain; hospinatmr@hotmail.com (N.M.-R.); cristinabaronadorado@gmail.com (C.B.D.); jmargo@odon.ucm.es (J.M.M.-G.); 3 Department of Conservative Dentistry and Orofacial Prosthodontics, Faculty of Dentistry, Complutense University of Madrid, 28040 Madrid, Spain; garciagil.ignacio@gmail.com

**Keywords:** marginal bone loss, dental implants, double acid-etched treated surface, probing depth, resonance frequency analysis

## Abstract

Background and objectives: The main purpose of this study was to evaluate the survival and success rates of dental implants with a double acid-etched surface treatment with evaluation times up to 10 years post-loading. *Materials and Methods:* This study was conducted at a hospital oral surgery and implantology unit. It included 111 dental implants with a double acid-etched surface. Three groups were created: Group 1 (1–3 years loading), Group 2 (3–5 years loading), and Group 3 (over 5 years loading). Probing depth, resonance frequency analysis (ISQ value), and marginal bone loss were evaluated. *Results:* The data obtained underwent statistical analysis. Overall, 78 patients were included in the study, who received, in total, 111 dental implants, all replacing single teeth. Mean probing depth was 3.03 mm and mean ISQ was 65.54. Regarding marginal bone loss, in Group 1, 67.6% of implants did not undergo any thread loss, in Group 2, 48.3%, and in Group 3, 59.6%; 59.10% of all implants did not present thread loss with a mean bone loss of 0.552 mm. The implant survival rate was 99.1%, and the success rate was 96.37%. *Conclusions:* Implants with a double acid-etched surface showed excellent success rates in terms of marginal bone loss, ISQ, and probing depth after up to 10 years of loading, making them a clinically predictable treatment option. Future studies are needed to compare this implant surface with other types in different restorative situations.

## 1. Introduction

Dental implant placement is one of the best treatment options for replacing one or more teeth. Treatment with implants has been extensively documented in the scientific literature [1,2,3]. The success of dental implants is based on osseointegration, among other principles. Osseointegration is the process of interaction between the surface of the dental implant and the patient’s bone. Therefore, the surface treatment of the dental implant—roughness, topography, and chemical composition—can increase the contact area between implant and bone, which improves osseointegration. Consequently, this point is considered to be one of the key factors for ensuring a high success rate [4,5,6,7,8,9,10].

Numerous types of surfaces have been described in the literature, all aiming to produce adequate osseointegration and bone-to-implant contact (BIC), while minimizing the incidence of mucositis and peri-implantitis [11]. One of these is the Avantblast^®^ surface; implants undergo surface modifications in three phases: the first is mechanical, in which particles impact on the surface; the second is treatment by a combined acid medium of hydrogen sulfide and hydrofluoric acid (double acid-etching); and the third is thermal treatment to stabilize and homogenize the titanium oxide surface layer [12]. Surfaces modified by thermal treatment improve bioactivity and bone-implant contact (BIC) and may achieve faster osteointegration. This can be a clinical advantage, especially for implants placed in areas with low-density bone [13]. 

Many studies support the use of this or similar implant surfaces due to the high implant survival rates obtained [12,14,15]. Nevertheless, it is important to evaluate other parameters affecting single teeth in the medium/long term, such as marginal bone loss (MBL), resonance frequency analysis (RFA, expressed as an ISQ value), and probing depth (PD). 

Implants and their surface treatments have been evaluated on multiple occasions by different research groups. Although much of the research in implantology focuses on the study of bioactive surfaces, there are very few clinical trials carried out in humans. These studies have compared the ISQ of two types of implants, with and without a bioactive surface [16]; another studied a series of titanium implants coated with (CaP) [17]; and a third study looked at the effectiveness of hydroxyapatite and bioactive glass-coated titanium implants [18]. 

A current systematic review of implant surfaces determines that the effect of bioactive modifications to dental implant surfaces is not always beneficial for osseointegration, although certain biomolecules used for veneering appear to influence early peri-implantation. Therefore, the results obtained using animal models cannot always be extrapolated to human clinical reality [19].

The aim of this study was to assess peri-implant mucosa behavior by analyzing: success and survival rates, ISQ implant stability, PD, and MBL around implants replacing single teeth, at different times up to 10 years post-loading.

## 2. Material and Methods

### 2.1. Study Design

**Figure 1:**
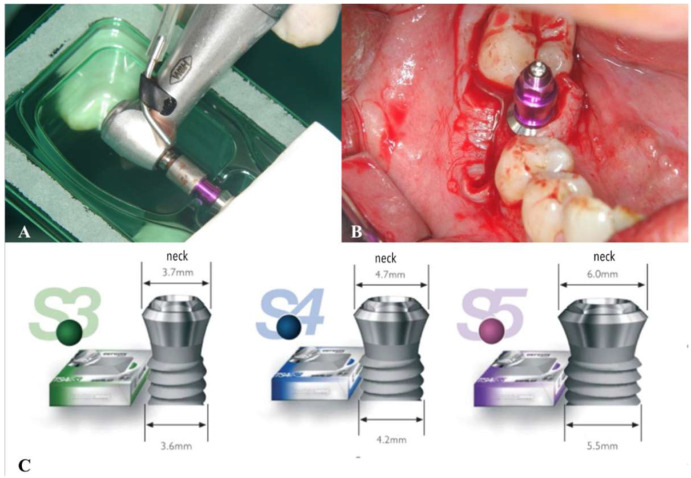
(**A**) Dental implant removed from packaging; (**B**) Implant placement in the mandibular posterior sector; (**C**) TSA dental implants of varying width.

Patients requiring replacement of a single tooth with an implant were included in the study; all attended an oral surgery service at the Virgen de la Paloma Hospital in Madrid. Patients were treated between July 2009 and July 2018 (Figure 1). 

The present study consists of all those patients who met the inclusion criteria during the recruitment period. A minimum sample size of 30 patients was established to be able to apply non-parametric tests with sufficient statistical relevance. At the statistical level, by the central limit theorem, it is assumed with a sample size N = 30 the sample presents a normal distribution of parameters Mu, Sigma: x N (u). 

**Figure 2:**
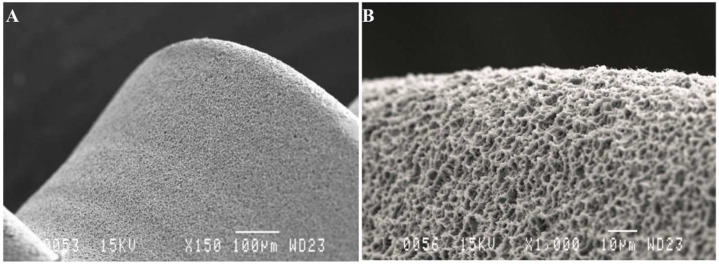
(**A**) SEM images provided by the manufacturer of the Avantblast^®^ surface (Phibo^®^ Dental Scheme 150 magnification); (**B**) Surface details ×1000 magnification.

All the dental implants analyzed were TSA (1.5 mm machined neck) with Avantblast^®^ surface (Phibo^®^ Dental System, Bacelona, Spain), which is treated with the triple process described above to create a rough surface (Sa 1.3 μm) (Figure 2).

The study was conducted according to the principles outlined in the Declaration of Helsinki for clinical research involving humans. The ethical committee approved the study protocol (nº 13/449-E), and all patients gave their informed consent to take part. Patients were provided with information about the clinical protocol, the purpose of the study, and all clinical and follow-up phases. 

Inclusion criteria were: patients aged 18 years or older; male/female; good systemic health status (ASA I or II) or patients with controlled chronic systemic diseases; sufficient bone width and height to allow dental implant placement (no bone regeneration needed); in patients with previous dental implants, all had been loaded for at least 1 year. Exclusion criteria were: women pregnant or lactating; presence of uncontrolled chronic systemic diseases (diabetes, cardiovascular diseases, or others); smoking > 10 cigarettes/day; alcohol or drug abuse.

Observation groups were based on two factors: implant loading time and implant location (Table 1). Three groups were established according to the time elapsed since loading: Group 1 (1–3 years loading); Group 2 (3–5 years loading); and Group 3 (over 5 years loading). 

A clinical protocol was created for each patient registering the following data: medical record number; age; sex; implant length; implant width; location: incisor/canine region, premolar or posterior region, subdividing between maxilla and mandible; PD; ISQ values; and MBL.

### 2.2. Study Parameters

**Figure 3:**
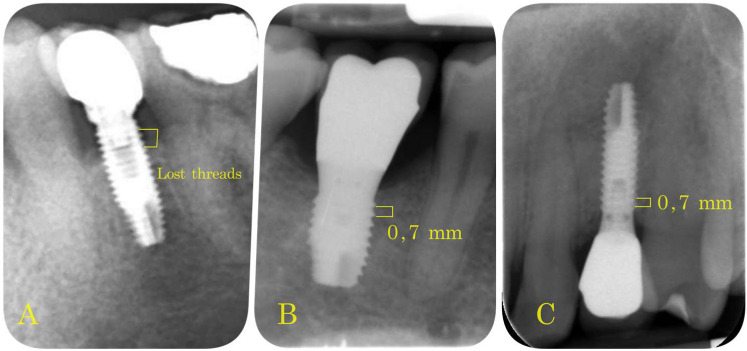
(**A**) Two lost threads in a mandible dental implant; (**B**) 0.7 mm represents the distance between threads (mandible dental implant); (**C**) 0.7 mm thread distance in a maxilla dental implant.

**Figure 4:**
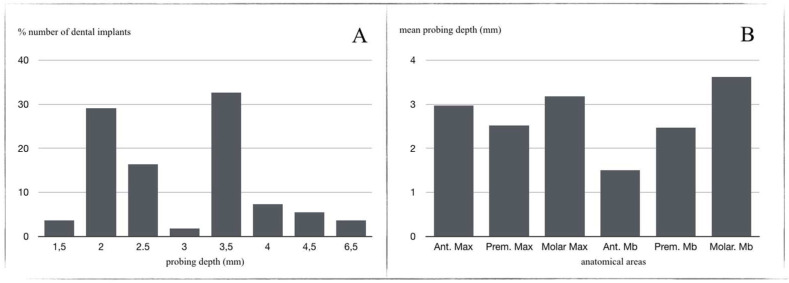
(**A**) Numbers of dental implants (%) according to PD (mm); (**B**) PD (mm) in relation to location (%).

The study analyzed 111 dental implants, all replacing single teeth. The following study variables were recorded to evaluate implant success in the medium-to-long term:

1. Probing Depth (PD): a periodontal probe cp-15 (Hu-Friedy^®^, Chicago, IL, USA), calibrated at intervals of 1 mm, was used to evaluate peri-implant mucosa behavior (Figure 3). PDs of 0–6 mm were considered physiological, and, when >6 mm, as pathological. Three points on vestibular and lingual/palatal aspects were evaluated for each implant;

2. To assess implant stability, resonance frequency analysis (RFA) produced an ISQ value for each implant. Evaluations were performed at different times after loading, depending on group. RFA was measured with the Ostell^®^ device (Malmö, Sweden), obtaining ISQ values on a scale of 1 to 100 (kHz). To obtain this, each patient’s implant-supported prosthesis was removed (prosthesis screwed to the implant in all cases) and implant stability was measured. The crown was then screwed back into the dental implant, applying a torque of 30 Ncm;
3. Marginal Bone Loss (MBL): periapical intraoral radiographs were used to evaluate bone loss around the implants (Sirona Heliodent with 1 mm aluminum filter). A Rinn Endoray^®^ plate holder (Markham, ON, Canada) was used to maintain the same object focus distance for all patients, and to avoid focus distance variations that could lead to inaccurate results. The distance between the bone crest and the implant shoulder on both mesial and distal aspects was measured from periapical radiographs by the same operator in all cases. These measurements were expressed by the number of implant threads lost (Figure 4).

### 2.3. Statistical Analysis

Statistical analysis of the data was carried out at the Data Processing Center of the Complutense University of Madrid (Spain), using the SPSS 24 statistical software package. Mean marginal bone loss, ISQ, and PD in each group were analyzed by means of ANOVA and chi-square tests. The level of significance was set at 5%. 

## 3. Results

The study population consisted of 78 patients, 48 females (61.5%) and 30 males (38.5%), establishing an F/M ratio of 1/0.63. The patients’ ages ranged from 23 to 75 years (mean age 50.85 years); age intervals were as follows: 23–35 years (11.54%); 36–50 years (20.51%); 51–65 years (53.85%); over 65 years (14.10%). Implant locations were distributed as follows: anterior sector (incisor-canine) (maxilla 20.9%; mandible 0.9%); premolar sector (premolars) (maxilla 36.4%; mandible 11.8%); and posterior sector (molars) (maxilla 5.5%; mandible 24.5%) (Table 1). 

Of 111 dental implants placed, only one implant failed two months before loading, making the survival rate 99.1%. 

Regarding probing depth, 96.37% of the implants had a probing depth equal to or less than 6 mm (1.5 mm (3.63%); 2 mm (29.09%); 2.5 mm (16.36%); 3 mm (1.815%); 3.5 mm (32.72%); 4 mm (7.27%); 4.5 mm (5.45%); 6.5 mm (3.63%)) (Figure 4a). Mean probing depth was 3.03 mm. When probing depth data were analyzed in relation to implant locations, it was found that implants in mandibular posterior regions presented the greatest probing depth, although the mean value did not exceed 6 mm: anterior sector (maxilla 2.973 mm; mandible 1.5 mm); premolar sector (maxilla 2.521 mm; mandible 2.467 mm); and posterior sector (maxilla 3.175 mm; mandible 3.615 mm) (Figure 4b). 

**Figure 5:**
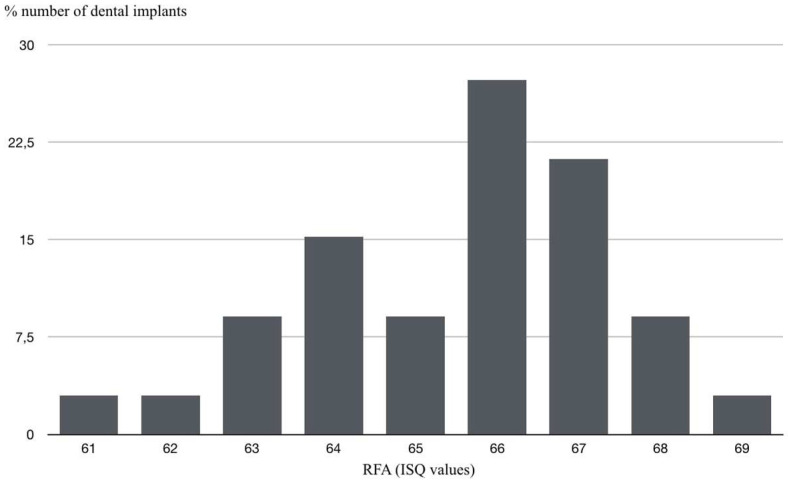
Numbers of dental implants (%) according to RFA (ISQ values).

The ISQ values showed a predominance of 66 in 27.3% of the implants, followed by 67 in 21.2% of cases. No values below 60 were obtained in any of the implants. Mean ISQ was 65.54 (Figure 5). A 95% statistically significant relation (*p* = 0.040) was observed between high ISQ values and low probing depths.

**Figure 6:**
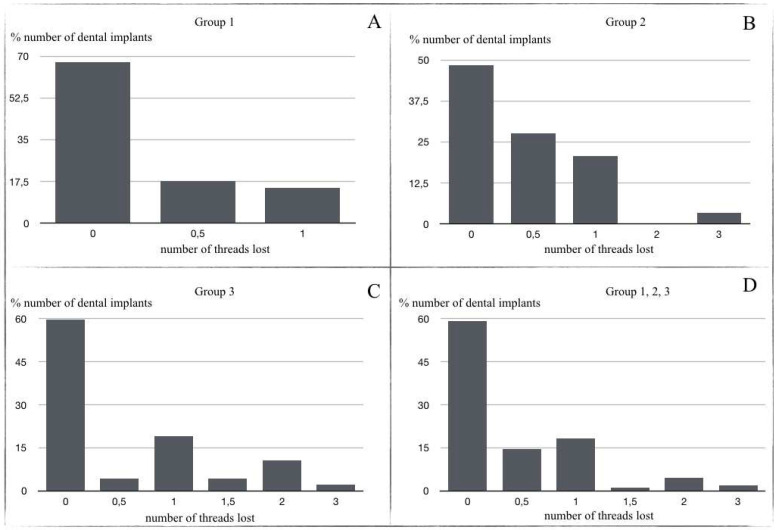
(**A**) Thread loss in Group 1 (after 1–3 years prosthetic loading); (**B**) Thread loss in Group 2 (after 3–5 years prosthetic loading); (**C**) Thread loss in Group 3 (up to 10 years prosthetic loading); (**D**) Mean thread loss in total sample.

Marginal bone loss was as follows: Group 1 (67.6% presented no thread loss (a single thread represents 0.7 mm); 17.6% 0.5 thread lost; and 14.7% 1 thread lost) (Figure 6a); Group 2 (48.3% presented no thread loss; 27.6% 0.5 thread lost; 20.7% 1 thread lost; 3.4% 3 threads lost) (Figure 6b); Group 3 (59.6% presented no thread loss; 4.3% 0.5 thread lost; 19.1% presented 1 thread lost; 4.3% 1.5 thread lost; 10.6% 2 threads lost; 2.1% 3 threads lost) (Figure 6c).

**Figure 7:**
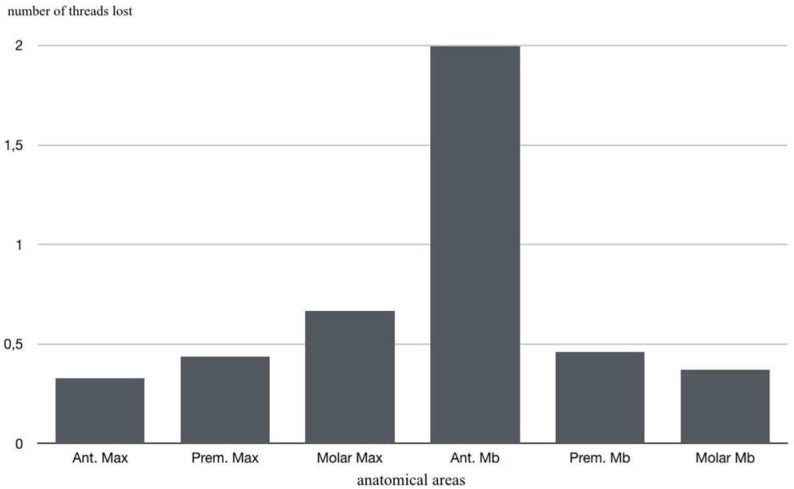
Mean thread loss in relation to implant location.

Analyzing all groups together, 59.10% of implants presented no thread loss; mean thread loss was 0.552 (0.38 mm) (Figure 6d). In relation to implant location, it was found that the greatest thread loss occurred in the mandibular anterior region with an average loss of two threads: anterior sector (maxilla 0.326; mandible 2); premolar sector (maxilla 0.438; mandible 0.462); and posterior sector (maxilla 0.667; mandible 0.37) (Figure 7).

## 4. Discussion

Dental implant placement to replace a missing tooth, whether in mandible or maxilla, has been shown to obtain high success rates and offers several advantages over other therapeutic options. However, dental implants may suffer biological and prosthodontic complications in the medium to long term [20]. For this reason, it is useful to determine which parameters are more or less relevant to the future survival and success of implants [21]. One of these is the implant surface, which influences marginal bone loss, implant stability, and probing depth, among other outcomes.

Implant surface roughness is a fundamental characteristic for ensuring early osseointegration and implant survival. The surface of dental implants can be treated in various ways to achieve this goal. Unfortunately, high surface roughness can trigger mucositis or peri-implantitis processes [22]. The present study obtained an excellent implant survival rate (99.1%), equal to or better than similar studies, for example Cochran et al. [23] who obtained 99.1% after 5 years (Table 2). Moreover, one systematic review of Jung et al. [24] established survival rates of 97.2% at 5 years and 95.2% at 10 years for titanium implants supporting single crowns.

Regarding marginal bone loss, it has been shown that several parameters influence bone levels around subcrestal implants. Of these, the type and height of the abutment used would appear to exert a significant influence on MBL [25,26]. However, the present study used tissue level implants, so this parameter may not have been very relevant to the MBL results [24], which were satisfactory (mean 0.38 mm), especially in posterior sectors. These results are consistent with other studies (although the present work used different MBL measurement techniques) including Calvo-Guirado et al. [27], who obtained a crestal bone loss 0.90 mm ± 0.26 mm with bone level dental implants after 5 years. 

One of the most relevant aspects when evaluating the survival and success of dental implants is the presence or absence of peri-implantitis, as the incidence of this pathology is increasing significantly [28]. In order to reduce the prevalence of this disease, the presence of peri-implant keratinized tissue and probing depth are parameters that are gaining importance in terms of dental implant maintenance [29,30,31]. In this context, probing depth is a key method for assessing the state of the peri-implant mucosa around implants, although this remains a controversial topic in the literature. Nevertheless, according to the consensus report issued at the last Workshop on the Classification of Periodontal and Peri-Implant Diseases and Conditions (2017), probing depths greater than 6 mm may be compatible with peri-implant disease when accompanied by bleeding upon probing and suppuration [32].

In the present study, PD was used as a diagnostic method obtaining a mean depth of 3.03 mm. This result is similar to Shi et al., who used tissue level dental implants (Straumann Bone Level, SLActive; Straumann AG, Basel, Switzerland) in a retrospective cohort study with up to 4 years follow-up, in which a screw-retained group (SG) obtained a mean PD of 3.5 mm (1.5–6.7) [33].

The success rate achieved in the present study was 96.37%, similar to other studies such as Froum et al. [34], who obtained almost the same success rate (96.4%) at 8.5 years with one-piece AOS dental implants (Nobel Direct, Nobel Biocare, Kloten, Switzerland), or Rammelsberg et al. [35] with a success rate of 97% for tissue-level and bone-level implants. Only 3.63% (*n* = 4) of the present sample obtained PDs greater than 6 mm; in two implants, this was accompanied by bleeding, and in the other two by suppuration on probing. 

The last parameter analyzed as relevant to dental implant survival was resonance frequency analysis [36]. This variable, expressed as ISQ values, has been shown to provide the surgeon with key information as to whether or not to carry out immediate loading after implant placement [37]. In addition to its usefulness in informing decision-making about loading at the moment of implant placement, ISQ values also act as a diagnostic measure of potential osseointegration failure and subsequent implant loss. Most studies obtain implant stability values of 60–70 ISQ regardless of the evaluation time [38,39]. Accordingly, Chen et al. [40] reported ISQ results at 5 years of 65.5 ± 6.90, which are similar to the present study (65.54). The present results also highlighted a 95% significant relation (*p* = 0.000) between marginal bone loss and lower ISQ values, as well as a 95% statistically significant relation (*p* = 0.040) between high ISQ values and low probing depths. 

All the parameters analyzed in the present work contribute to determining the long-term outcome of any treatment involving one or more dental implants. In this context, there are a range of factors that every clinician must bear in mind in order to achieve implant survival and success, and to avoid peri-implant pathology, which is becoming increasingly prevalent [41]. Some of these factors are specific to the individual patient (age, smoking, oral hygiene, systemic disease, etc.), and others to the treatment itself (implant type, macroscopic implant design, implant position, the type of abutment used, etc.); all will have a direct impact on treatment outcomes [42]. In the present study, the implant’s surface was considered a differentiating characteristic of central importance for obtaining adequate success rates in terms of MBL, PD, and implant stability. The implant surface selected for the present investigation has been shown to achieve success rates similar to other surfaces with the same or similar characteristics over periods of 1 to 10 years post-loading, making it possible to compare the present results with previous studies. Nevertheless, in future research it would be interesting to compare different types of surface in the medium to long term, not only implants used to support single crowns, but implants supporting other types of restorations.

There are many studies that have included multiple elements in surface treatment such as propolis or other substances; these present a series of limitations, especially due to the difference in the measurement of the clinical parameters of periodontal disease and peri-implantitis, such as the level of plaque, the periodontal indices analyzed, or the importance of saliva [43].

The inflammatory response is a determining factor in the success of implants taking into account that the so-called tribocorrosion process releases titanium ions into the surrounding tissues, which can trigger a cascade of reactions, localized or at distance, or even systemic reactions [44]. In this sense, the importance of the treatment of the implant surfaces, and the surface treatment of the prosthetic attachments and their influence on the behavior at the mucosal level, has been evaluated by different authors, concluding that the direct metal laser sintered healing abutments (DMLS) promote the decrease in the subgingival inflammatory infiltrate, and therefore better adherence of the mucosa to the prosthetic pilar than the existing union by integrins of the biological tissue [45]. Other authors have not found significant differences in the count of infiltrated T and B lymphocytes, IL-17, and RANKL expressions when studying the cellular and molecular patterns of bone loss in the soft tissue surrounding implants restored with different platform configurations implant [46]. 

Finally, it should be noted that other materials, such as ceramic materials [47] or bioactive glass fiber reinforced composite (GFRC) implants [48], have been proposed recently as potential alternatives to titanium implants. The quest for improved esthetics and apprehension about titanium hypersensitivity have led to increasing demand for these substitute materials [47].

## 5. Conclusions

This medium- to long-term follow-up, observational study of dental implants with double acid-etched surface (Avantblast^®^) obtained excellent survival and success rates when replacing single teeth. The survival rate was 99.1% and the success rate 96.37%. ISQ values pointed to adequate osseointegration at up to 10 years’ post-loading, while evaluations of marginal bone loss and probing depth obtained acceptable results at all follow-up times.

Within the limitations of this study, it may be concluded that implants with double acid-etched treated surface constitute a clinically predictable treatment option. Further studies are needed to compare the behavior of different implant surfaces in a range of restorative situations. 

## Figures and Tables

**Table 1:** Distribution of the sample in relation to diameter, length, time of loading, and implant location.

Implant Characteristics		*n* = 110		
Diameter	S3 (3.6 mm) nº implants: 30 (27%)	S4 (4.2 mm) nº implants: 50 (46%)	S5 (5.5 mm) nº implants: 30 (27%)	
Length	10 mm nº implants: 28 (25.5%)	11.5 mm nº implants: 34 (30.9%)	13 mm nº implants: 36 (32.7%)	14.5 mm nº implants: 12 (10.9%)
	anterior sector (incisor-canine)	premolar sector (premolars)	posterior sector (molars)	
Location	nº maxilla: 23 (20.9%) nºmandible: 1 (0.9%)	nº maxilla: 39 (35.5%) nº mandible: 13 (11.8%)	nº maxilla: 6 (5.5%) nº mandible: 28 (25.5%)	
Time of loading	from 1 to 3 years nº implants: 28 (25.2%)	from 3 to 5 years nº implants: 70 (63.96%)	more than 5 years nº implants: 12 (10.81%)	

**Table 2:** Results of this study in comparation with other similar studies of the literature.

Authors	Year	Study Type	Nº Patients	Age (Years)	Nº Implants	Implant System	MBL (mm)
Cochran et al. [23]	2011	Prospective multicenter	135	55	*n* = 439	St SLA	NR
Rammelsberg et al. [35]	2017	Prospective	630	59.56	*n* = 1569	St TL, St BL, Nb Replace	NR
Froum et al. [34]	2017	Cohorts	28	56.8y	*n* = 28	Nb	0.848
Shi et al. [33]	2018	Retrospective cohort	176	49.6 SG; 46.8 CG	*n* = 176	St TL	3.5
Calvo-Guirado et al. [27]	2018	Prospective	53	37.85	*n* = 71	MIS	0.90
Chen et al. [40]	2019	Retrospective analysis	173	21–85	*n* = 383	NR	0.03 ± 0.091
Our study	2021	Observational	110	50.85	*n* = 111	Phibo^®^ TSA	0.38
**Authors**	**RFA (mean ISQ)**	**Probing depth (mm)**	**Survival rate (%)**	**Success rate (%)**	**Restoration**	**follow-up (years)**	
Cochran et al. [23]	NR	2.7	99.1	98.8	NR	5	
Rammelsberg et al. [35]	NR	NR	NR	97 (single crowns)	NR	5.0	
Froum et al. [34]	NR	2.089	100	96.4	NR	8.5	
Shi et al. [33]	NR	3.78(SG); 3.43(CG)	100 SG, 98.8 CG	NR	CG, SG	2.5	
Calvo-Guirado et al. [27]	NR	NR	100 %	NR	CG, SG	5	
Chen et al. [40]	65.5 ± 6.9	NR	95		NR	10	
Our study	65.54	3.4 mm	99.1	96.37	SG	From 1 to 10 years	

St = Straumann; Nb = Nobel Biocare; BL = Bone Level; TL = Tissue Level; Cemented implant crown Group (CG); Screw implant crown group (SG).

## Abbreviations

BIC	Bone Implant Contact
MBL	Marginal Bone Loss
RFA	Resonance frequency analysis
ISQ	Implant stability quotient
PD	Probing Depth
